# ﻿Rediscovery of *Lycodongammiei* (Blanford, 1878) (Serpentes, Colubridae) in Xizang, China, with comments on its systematic position

**DOI:** 10.3897/zookeys.1200.117260

**Published:** 2024-05-07

**Authors:** Fu Shu, Bing Lyu, Keji Guo, Tong Zhang, Xiaoqi Mi, Li Li, Yayong Wu, Peng Guo

**Affiliations:** 1 Central South Academy of Inventory and Planning of National Forestry and Grassland Administration, Changsha 410014, China Central South Academy of Inventory and Planning of National Forestry and Grassland Administration Changsha China; 2 Faculty of Agriculture, Forestry and Food Engineering, Yibin University, Yibin 644005, China Yibin University Yibin China; 3 College of Agriculture and Forestry Engineering and Planning, Guizhou Provincial Key Laboratory of Biodiversity Conservation and Utilization in the Fanjing Mountain Region, Tongren University, Tongren 554300, Guizhou, China Tongren University Tongren China

**Keywords:** Himalayas, phylogeny, Qinghai-Xizang Plateau, snake

## Abstract

*Lycodongammiei* (Blanford, 1878), a rarely encountered species of Asian snake, is characterized by ambiguous systematics and biology. Based on a sole specimen of *L.gammiei* rediscovered in southeastern Xizang, China, we conduct a detailed morphological examination and description, and investigate the systematic position of this species. Morphologically, the newly collected specimen is closely aligned with specimens previously described. Mitochondrial DNA-based phylogenetic analyses reveal that *L.gammiei* constitutes an independent evolutionary lineage, forming a clade with *L.fasciatus* (Anderson, 1879), *L.gongshan* Vogel & Luo, 2011, *L.butleri* Boulenger, 1900, and *L.cavernicolus* Grismer, Quah, Anuar, Muin, Wood & Nor, 2014. The closest genetic distance between *L.gammiei* and its congeners was 10.2%. The discovery of *L.gammiei* in Medog County, China, signifies an eastward expansion of its known geographical distribution.

## ﻿Introduction

Exploring the boundaries of geographic distribution and systematic position of species is crucial for understanding their evolutionary origins and diversification and for devising appropriate conservation strategies. Despite considerable progress in recent years, many species remain poorly known and explored. This is particularly evident for some snake species due to their rarity and cryptic habitats.

*Lycodongammiei* (Blanford, 1878), a rare non-venomous snake species within the family Colubridae, was initially described as *Ophitesgammiei* based on a single specimen collected from Darjeeling, West Bengal, India (Blanford 1878). Subsequently, it was reclassified into the genus *Lycodon* (Boulenger 1890) or *Dinodon* (Wall 1923; Smith 1943), identifying it as *Lycodongammiei*. Wall (1911) compared *L.gammiei* and *L.fasciatus* (Anderson, 1879), and he synonymized *L.fasciatus* with *L.gammiei*. However, Wall (1923) later revised this view, recognizing its distinctiveness and validity of *L.fasciatus*. Mahendra (1984) proposed that *L.gammiei* was a color variety of *L.septentrionalis* (Gunther, 1875), while this synonymy was not accepted by all authors. Since its initial description, *L.gammiei* has been found in southeastern Xizang, China (Agarwal et al. 2010) and in Bhutan (Wangyal 2013). To date, however, few specimens of the species have been collected, and no genetic data have been reported.

In 2023, we collected a living specimen of *L.gammiei* in Medog County, southeastern Xizang, China. The rediscovery of this species in Xizang not only extends this species’ geographic distribution but also allows the exploration of its systematic position through molecular data.

## ﻿Materials and methods

### ﻿Morphological examination

**Figure 1.**
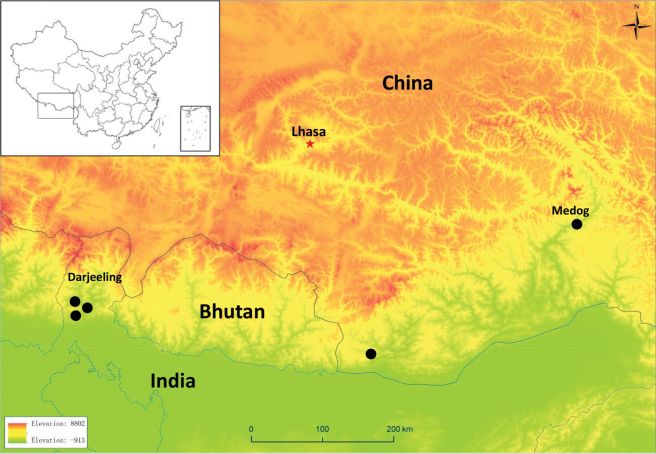
Map showing currently known localities of *Lycodongammiei*.

The specimen deposited at Yibin University (YBU 230088) was collected in Beibeng Town, Medog County, southeastern Xizang, China (29°14′02″N, 95°10′38″E) (Fig. 1) on 14 August 2023 at an elevation of 1,431 m by Xiaoqi Mi. The snake was found on a tree near a road at 23:30 hours. Characters relating to scalation, color pattern, and body proportions were recorded from the preserved specimen in laboratory. Snout–vent length (SVL) and tail length (TL) were measured using a meter ruler to the nearest 0.5 centimeter, while all remaining measurements were taken using digital calipers to the nearest millimeter. Symmetric mensural head characters were taken on the right side unless unavailable (e.g. damaged), while meristic characters were recorded on both sides and reported in left/right order.

Comparative data of other specimens of this species were taken from the literature (Blanford 1878; Mistry et al. 2007; Chettri and Bhupathy 2009; Wangyal 2013).

### ﻿Molecular phylogeny

Genomic DNA was extracted from the liver tissue of the newly collected specimen using an Animal Genomic DNA Purification Kit (TIANGEN Bio-tech Co., Ltd, Beijing, China). Subsequently, a fragment of the mitochondrial gene cytochrome b (cyt b) was amplified using primers H14919 (5′-AACCACCGTTGTTATTCAACT-3′) and L16064 (5′-CTTTGGTTTACAAGAACAATGCTTTA-3′) (Burbrink et al. 2000). The polymerase chain reaction (PCR) products were purified and sequenced in both directions by Sangon Biotech Co., Ltd (Chengdu, China). The obtained sequences were manually edited using SeqMan in Lasergene v. 7.1 (DNASTAR, USA), and aligned using the ClustalW algorithm with default parameters in MEGA v. 7.0 (Kumar et al. 2016), followed by a visual inspection for minor manual adjustments. The DNA sequences were translated into amino acid sequences using MEGA v. 7.0 to verify sequence quality and detect any unexpected stop codons (Kumar et al. 2016). Furthermore, 80 additional sequences were downloaded from GenBank (Table 1).

**Table 1.** Detail information for the samples used in this study.

No.	Species	Voucher Number	Locality	GenBank No.
1	*Lycodonalbofuscus*	LSUHC 3867	–	KX660500
2	*Lycodonalbofuscus*	LSUHC 4588	–	KX660501
3	*Lycodonalcalai*	KU 327847	Barangay San Antonio, Batanes Province, Philippines	KC010344
4	*Lycodonalcalai*	KU 327848	Municipality of Sabtang, Batanes, Philippines	KC010345
5	*Lycodonanakradaya*	SIEZC 20247	Song Giang River, Khanh Hoa Province, Vietnam	OM674283
6	*Lycodonanakradaya*	SIEZC 20248	Song Giang River, Khanh Hoa Province, Vietnam	OM674284
7	*Lycodonaulicus*	KU 315378	Tablas Island, Romblon Province, Philippines	KC010350
8	*Lycodonaulicus*	PNM 7705	Leyte Island, Leyte province, Philippines	KC010349
9	*Lycodonbanksi*	VNUF R2015.20	Khammouane, Laos	MH669272
10	*Lycodonbibonius*	KU 304589	Cagayan, Philippines	KC010351
11	*Lycodonbutleri*	LSUHC 8365	Perak, Malaysia	KJ607892
12	*Lycodonbutleri*	LSUHC 9137	Perak, Malaysia	KJ607891
13	*Lycodoncapucinus*	–	–	MK844525
14	*Lycodoncapucinus*	MVZ 291703	Timor	MK844522
15	*Lycodoncapucinus*	MVZ 291704	Timor	MK844523
16	*Lycodoncathaya*	SYS r001542	Longsheng County, Guangxi, China	MT602075
17	*Lycodoncathaya*	SYS r001630	Longsheng County, Guangxi, China	MT602076
18	*Lycodoncavernicolus*	LSUHC 10500	Perlis, Malaysia	KJ607890
19	*Lycodoncavernicolus*	LSUHC 9985	Perlis, Malaysia	KJ607889
20	Lycodoncf.flavozonatus	KIZ 032400	Zayu, Xizang, China	MW199792
21	*Lycodonchapaensis*	KIZ 27593	Tengchong, Yunnan, China	MW353741
22	*Lycodonchapaensis*	KIZ 35013	Lushui, Yunnan, China	MW353742
23	*Lycodonchrysoprateros*	KU 307720	Cagayan, Philippines	KC010360
24	*Lycodondeccanensis*	–	Tumkur District, Karnataka, India	MW006487
25	*Lycodondeccanensis*	NCBS NRC AA0010	Karnataka, India	MW006486
26	*Lycodondumerilii*	KU 305168	Dinagat Island, Philippines	KC010362
27	*Lycodondumerilii*	KU 319989	Mindanao Island, Agusan del Sur Province, Philippines	KC010361
28	*Lycodondumerilii*	PNM 7751	Leyte Island, Leyte Province, Philippines	KC010363
29	*Lycodoneffraenis*	KU 328526	Karome, Nakhon Si Thammarat, Thailand	KC010364
30	*Lycodoneffraenis*	LSUHC 9670	Kedah, West Malaysia	KC010376
31	*Lycodonfasciatus*	CHS 837	Yunnan, China	MK201559
32	*Lycodonfasciatus*	KIZ 46120	Himalayan region in China	MW111468
33	*Lycodonflavicollis*	–	Devarayanadurga, Karnataka, India	MW006488
34	*Lycodonflavicollis*	AIWC 081	India	MZ029434
35	*Lycodonflavozonatus*	KIZ 023279	Xizang, China	MW199789
36	*Lycodonflavozonatus*	KIZ 07067	Xizang, China	MW199790
37	*Lycodonfutsingensis*	CHS 670	Nankunshan, Guangdong, China	MK201463
38	*Lycodonfutsingensis*	CHS 751	Guangdong, China	MK201504
39	*Lycodongammiei*	YBU 230088	Medog, Xizang, China	**OR842906**
40	*Lycodongongshan*	GP 3547	Lingcang,Yunnan, China	KP901025
41	*Lycodongongshan*	GP 3548	Lingcang,Yunnan, China	KP901026
42	*Lycodonjara*	CAS 235387	Putao, Kachin, Myanmar	KC010367
43	*Lycodonlaoensis*	FMNH 258659	Salavan, Laos	KC010368
44	*Lycodonlaoensis*	LSUHC 8481	O’Lakmeas, Pursat Province, Cambodia	KC010370
45	*Lycodonliuchengchaoi*	CHS 158	Sanjiazhai, Yunnan, China	MK201303
46	*Lycodonliuchengchaoi*	CHS 843	Ningshan, Shaanxi, China	MK201563
47	*Lycodonliuchengchaoi*	CHS 873	Shennongjia, Hubei, China	MK201580
48	*Lycodonmackinnoni*	ADR 197	Dhobighat, BWLS, Mussoorie, Uttarakhand	MW862977
49	*Lycodonmeridionalis*	CHS 870	Hechi, Guangxi, China	MK201578
50	*Lycodonmeridionalis*	VNUF R2012.4	Bac Kan, Vietnam	MH669271
51	*Lycodonmeridionalis*	VNUF R2017.123	Thanh Hoa Province, Vietnam	MH669270
52	*Lycodonmuelleri*	DLSUD 031	Luzon Island, Cavite Province, Philippines	KC010373
53	*Lycodonmuelleri*	KU 313891	Luzon Island, Camarines Norte Province, Philippines	KC010375
54	*Lycodonmuelleri*	KU 323384	Luzon Island, Aurora Province, Philippines	KC010374
55	*Lycodonnamdongensis*	VNUF R2017.23	ThanhHoa, Vietnam	MK585007
56	*Lycodonobvelatus*	KIZ 040146	Panzhihua, Sichuan, China	MW353745
57	*Lycodonpictus*	CIB 115609	Longzhou, Guangxi, China	MT845095
58	*Lycodonpictus*	VNMN 011227	Ha Lang, Cao Bang, Vietnam,	MT845094
59	*Lycodonrosozonatus*	CHS 794	Jianfengling, Hainan, China	MK201531
60	*Lycodonrufozonatus*	CHS 601	Huangshan, Anhui, China	MK201427
61	*Lycodonrufozonatus*	CHS 710	Yingpanxu, Hunan, China	MK201482
62	*Lycodonruhstrati*	CHS 776	Guangxi, China	MK201521
63	*Lycodonruhstrati*	CHS 803	Huaping, Guangxi, China	MK201538
64	*Lycodonsemicarinatus*	KUZJPN 28044	–	LC640371
65	*Lycodonseptentrionalis*	CHS 162	Yunnan, China	MK201305
66	*Lycodonseptentrionalis*	KIZ 46117	Xizang, China	MW199801
67	*Lycodonserratus*	KIZ 038335	Deqin, Yunnan, China	MW353746
68	*Lycodonstormi*	JAM 7487	Air Terjun Moramo, Sulawesi, Indonesia	KC010380
69	*Lycodonstriatus*	–	Savandurga, Karnataka, India	MW006489
70	*Lycodonstriatus*	CUHC 10368	Pakistan	OQ282988
71	*Lycodonstriatus*	CUHC 11257	–	OQ282989
72	*Lycodonstriatus*	CUHC 9457	–	OQ282987
73	*Lycodonsubcinctus*	CHS 734	Guangdong, China	MK201493
74	*Lycodonsubcinctus*	CHS 797	Diaoluoshan Mountain, Hainan, China	MK201534
75	*Lycodonsynaptor*	GP 3515	Lingcang, Yunnan, China	KP901021
76	*Lycodonsynaptor*	KIZ 046953	Xizang, China	MW199805
77	*Lycodontruongi*	SIEZC 20249	Song Giang River, Khanh Hoa Province, Vietnam	OM674282
78	*Lycodonzawi*	CAS 210323	Thabakesay, Saging, Myanmar	AF471040
79	*Lycodonzawi*	CAS 239944	Kyaukpyu, RakhineState, Myanmar	KC010386
80	*Lycodonzayuensis*	GP 7327	Zayu, Xizang, China	OP434398
81	*Lycodonzayuensis*	GP 7329	Zayu, Xizang, China	OP434399

Both Bayesian-inference (BI) and maximum-likelihood (ML) analyses were executed for the final dataset. Prior to analyses, the best-fit model of nucleotide substitution was selected for each partition (codon position) using Akaike Information Criterion (AIC) implemented in PartitionFinder (Lanfear et al. 2012). The BI analyses were conducted using MrBayes v. 3.2.2 (Ronquist et al. 2012). Searches consisted of three independent runs, each involving four Markov chains (three heated chains and one cold chain), with 10 million generations, sampling every 2,000 generations and with 25% of initial samples discarded as burn-in. Convergence was determined via effective sample size (ESS > 200) and likelihood plots against time using Tracer v. 1.7 (Rambaut et al. 2018). The resulting trees were combined to determine the posterior probabilities (PP) for each node based on a 50% majority-rule consensus tree. The ML trees were constructed in IQ-tree (Lam-Tung et al. 2015) using the GTRCAT model and the same partitioning scheme. In total, 1,000 Ultrafast bootstraps (UFB) topological replicates were performed for branch support assessment. *Boigacynodon* (Boie, 1827) was selected as the outgroup following previous research (Guo et al. 2013).

Uncorrected genetic distance (*p*-distance) was calculated in MEGA v. 7.0 (Kumar et al. 2016).

## ﻿Results

### ﻿Morphological description

Female, SVL 698 mm and TL 223 mm. Body elongated; head rather flattened; snout blunt. Rostral large, trapezoid; internasals much broader than long; prefrontals 3.0 mm in length, distinctly wider than long, extending beyond both sides and touching preocular and loreal; frontal peltate, 4.6 mm in length and 4.1 mm in width; parietals subrectangular, 7.9 mm in length and 4.2 mm in width. Nasals large, nostril located anteriorly and opening backward; loreal scale 1, long, nearly rectangular, failing to touch eye; preocular 1, postoculars 2; temporals 2+2+3. Supralabials 8, 1^st^ small, 3^rd^, 4^th^, and 5^th^ entering orbit, 6^th^ highest, 7^th^ largest; infralabials 10, first pair in contact, 1^st^ to 5^th^ in contact with anterior chin shields. Chin shield pairs 2, elongate, anterior pair slightly larger than latter pair. Dorsal scales 17-17-15 rows, scales weakly keeled, except for outermost several rows; scales reduced from 17 to 15 at 143^rd^ ventral position. Ventrals 228 (+ 1 preventral); cloacal plate entire; subcaudals 106, paired, dorsal scales of the tail reduced from 6 to 4 at 16^th^ subcaudal position.

**Figure 2.**
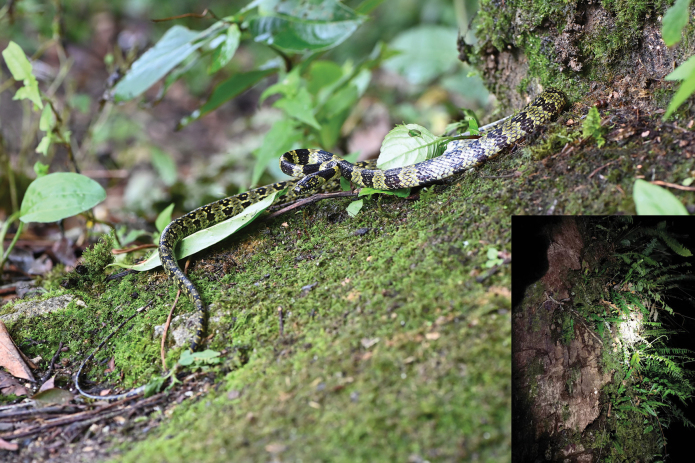
General view of the studied specimen (YBU 230088) in life and its microhabitat a big tree trunk (by XQ Mi).

**Figure 3.**
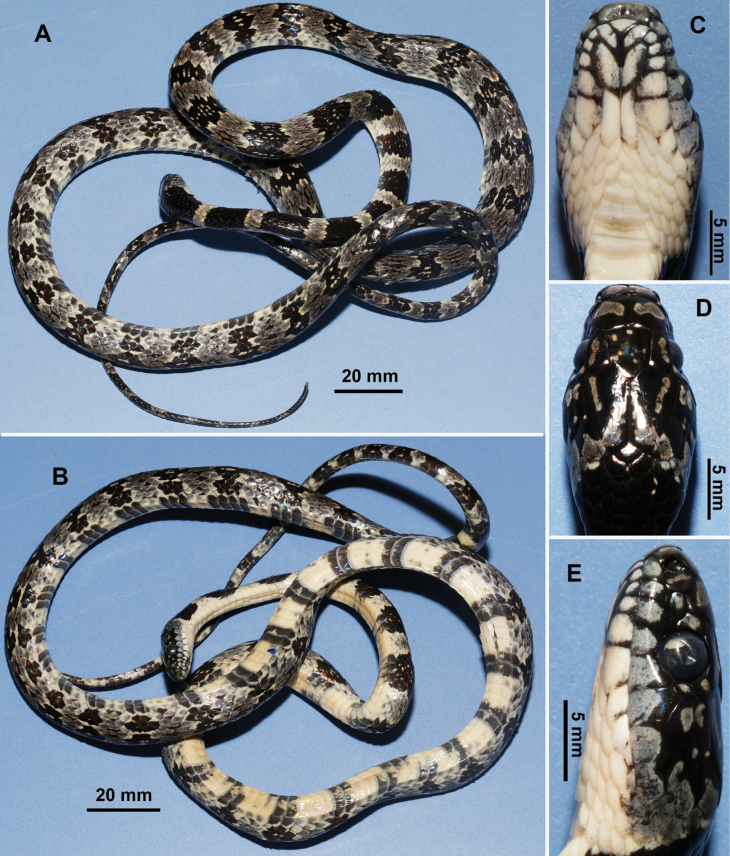
Views of the studied specimen (YBU 230088) in preservation. General dorsal (**A**) and ventral (**B**) views of specimen, dorsal (**C**), ventral (**D**) and lateral (**E**) views of head (by P Guo).

Head black, with yellow spots or short lines on some shields. Large, yellow spots on each side of posterior part of head. Conspicuous yellow collar on neck. Supralabials and anterior infralabials light yellow with dusky margins. Body surrounded by alternating dusky and light-yellow rings with very irregular, crooked margins. Yellow rings on body totaling 43, first pale ring clear above, anterior dark patch not continuous across throat, remaining rings encircling body. Lower part of head and neck light yellow. On belly, across anterior part of body, dark rings only about half as broad as light-yellow rings, less difference above, dark rings near head much broader above than white rings. Yellow rings on tail totaling 21 (Fig. 2). Preserved specimen somewhat faded, with no yellow visible (Fig. 3).

### ﻿Molecular phylogeny

In total, 1,047 bp of sequence data from 84 samples were aligned, with the generated novel sequence deposited in GenBank (Table 1). No deletions, insertions, or stop codons were detected, indicating that unintentional amplification of pseudogenes was unlikely (Zhang and Hewitt 1996). The best-fit evolutionary models of the data were: GTR+I+G for the first codon position, HKY+I+G for the second codon position, and GTR+G for the third codon position.

**Figure 4.**
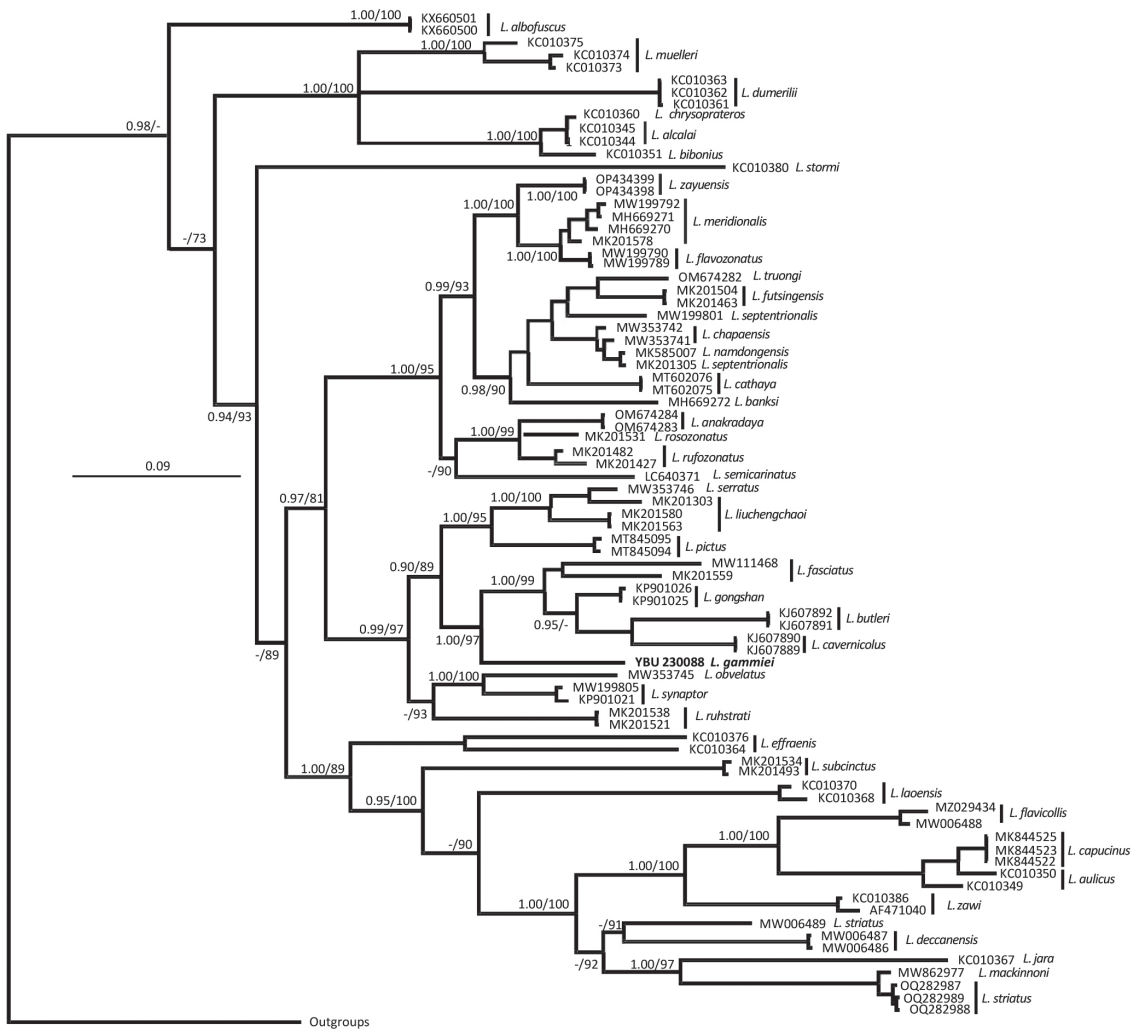
Bayesian 50% majority-rule consensus tree of *Lycodon* inferred from cyt b sequences analyzed using models detailed in the text. Posterior probabilities from BI analysis (>0.50) and Ultrafast bootstraps from ML analysis (>50%) are given adjacent to respective nodes for major nodes. Branch support indices are not given for most nodes to preserve clarity.

The mtDNA-based BI and ML analyses depicted relatively consistent topologies, with slight disagreement in several shallow nodes (Fig. 4). Both analyses indicated that all putative species of *Lycodon* formed a highly supported lineage (100 PP and 84% UFB). The newly collected specimen formed a clade with *L.fasciatus*, *L.gongshan* Vogel & Luo, 2011, *L.butleri* Boulenger, 1900, and *L.cavernicolus* Grismer, Quah, Anuar, Muin, Wood & Nor, 2014 with high support (100 PP and 97% UFB). Nevertheless, it occupied a basal position in relation to this clade and did not exhibit monophyly with any individual member. Uncorrected *p*-distances among the species within this clade ranged from 7.2% (*L.gongshan* and *L.fasciatus*) to 12.9% (*L.gammiei* and *L.cavernicolus*), while genetic distances between *L.gammiei* and its congeners within this clade ranged from 10.2% to 12.9% (data not shown).

## ﻿Discussion

*Lycodongammiei* is an exceedingly rare species, with a global record of only approximately 10 specimens. The majority of these are from Sikkim and West Bengal, India (Mistry et al. 2007; Chettri and Bhupathy 2009), with only two specimens reported in Cona County, Xizang, China (originally recorded in Eaglenest Wildlife Sanctuary, India) (Mistry et al. 2007) and Bhutan (Wangyal 2013), respectively. Based on the record by Mistry et al. (2007), Luo et al. (2010) recognized the existence of this species in China, although this recognition has been overlooked in subsequent publications (Wallach et al. 2014; Wang et al. 2020; Uetz et al. 2024). The discovery of this species in Medog County, Xizang, China, not only confirms its presence in China but also indicates a further eastward extension of its distribution.

Morphologically, the newly collected specimen shares most characters with the other conspecific specimens (Blanford 1878; Mistry et al. 2007; Chettri and Bhupathy 2009), including eight supralabials (3^rd^ to 5^th^ touching eye, 6^th^ largest), single loreal, 2+3 temporals, one preocular, two postoculars, two genial pairs, cloacal plate entire, and dorsal scales in 17-17-15 rows. However, the new specimen has a greater number of ventral scales (228+1) than all previously reported specimens (205–220) (Mistry et al. 2007; Chettri and Bhupathy 2009).

The taxonomic status of *L.gammiei* has a controversial history. Although previously misidentified as both *L.fasciatus* (Wall 1911) and *L.septentrionalis* (Mahendra 1984), Mistry et al. (2007) later clarified its distinct status and validity based on morphological comparisons. In the current study, we present the first genetic data pertaining to this species. Notably, mtDNA-based phylogenetic analyses indicated that *L.gammiei* formed a highly supported monophyly with a clade containing *L.fasciatus* but was not the closest congener to *L.fasciatus* within this assemblage (Fig. 4). *Lycodongammiei* shows a greater genetic distance from *L.septentrionalis* than from *L.fasciatus*, further affirming its validity and unique taxonomic position. The closer genetic affinity of *L.gammiei* with the clade encompassing *L.fasciatus* aligns with their geographical closeness along the southern slopes of the Himalayas.

*Lycodonzayuensis* Jiang, Wang, Jin & Che, 2020 coexists with *L.gammiei* in southeastern Xizang, China (Che et al. 2020; Lyu et al. 2022). Both species exhibit similarities in external morphology, including dorsal scales in 17-17-15 rows, eight supralabials, one preocular, and two postoculars. However, the two species are genetically divergent (Fig. 2), and *L.gammiei* can be easily distinguished from *L.zayuensis* by its broader and fewer yellow body cross-bands (30–43 vs 88–93) (Blanford 1878; Lyu et al. 2022).

## ﻿Additional information

### Author ORCIDs

Fu Shu https://orcid.org/0000-0002-6082-8112

Bing Lyu https://orcid.org/0000-0001-5594-1543

Keji Guo https://orcid.org/0000-0001-7508-1173

Tong Zhang https://orcid.org/0009-0009-1492-585X

Xiaoqi Mi https://orcid.org/0000-0003-1744-3855

Li Li https://orcid.org/0009-0007-4149-6662

Yayong Wu https://orcid.org/0000-0003-2752-4085

Peng Guo https://orcid.org/0000-0001-5585-292X

### Data availability

All of the data that support the findings of this study are available in the main text.
